# Microstructure and Mechanical Behavior of Modern Universal-Chromatic and Bulk-Fill Resin-Based Composites Developed to Simplify Dental Restorative Procedures

**DOI:** 10.3390/jfb13040178

**Published:** 2022-10-07

**Authors:** Nicoleta Ilie, Marioara Moldovan, Andrei C. Ionescu

**Affiliations:** 1 Department of Conservative Dentistry and Periodontology, University Hospital, Ludwig-Maximilians-University, Goethestr. 70, 80336 Munich, Germany; 2 Institute of Chemistry Raluca Ripan, Babes-Bolyai University, 30 Fantanele Street, 400294 Cluj-Napoca, Romania; 3 Oral Microbiology and Biomaterials Laboratory, Department of Biomedical, Surgical and Dental Sciences, Università Degli Studi di Milano, Via Pascal 36, 20133 Milano, Italy

**Keywords:** resin-based composites, 3-point bending, structural-coloring, viscoelastic, quasi-static, DMA, aging

## Abstract

One of the recent trends in the development of resin-based composites (RBCs) focuses on universal coloring to avoid time-consuming color matching and RBC layering for a clinically appropriate esthetic impact. We evaluated an experimental material for posterior restorations combining universal coloring with the possibility of bulk-fill placement. Clinically established materials were analyzed as a reference, including a bulk-fill and a universal chromatic RBC. Microstructural features were described using scanning electron microscopy and related to macroscopic and microscopic mechanical behavior. Standards to be met before market launch were supplemented by fractography, Weibull analysis, and aging behavior assessment. Quasi-static and viscoelastic behavior were evaluated on a microscopic scale, incorporating a large number of parameters and increasingly aggressive immersion media. All materials complied with the standard requirements even after aging. The latter had little impact on the measured parameters, except for strength. Strength, modulus of elasticity, and hardness parameters on the one hand and damping behavior on the other were mutually exclusive. Despite considerable differences in the microstructure and type of filler, an increased filler amount remained critical for better mechanical properties. The lower proportion of inorganic fillers was directly transferred to the elastic modulus values, which, in turn, restricts the experimental material in its clinical applications to smaller occlusal fillings.

## 1. Introduction

Modern dentistry continuously strives to develop innovative restoration concepts to simplify, shorten, and improve the predictability of a restorative treatment. In this context, increasingly complex materials are being developed to reduce the number of treatment steps or facilitate the selection of suitable materials. Resin-based universal chromatic composites (RBC-UC) are one of these new developments that allow to achieve highly aesthetic outcomes using only a single shade, independent of the color of the underlying and adjacent tooth structure. In RBC-UC, this trend is essentially being pursued by two different philosophies.

The conventional technology for coloring dental RBCs is light absorption due to suitable pigments. Universal coloring in most RBC-UC brands appears to use the same technology, as materials contain filler systems similar to regular RBCs [1,2]. The similarity in filler systems has recently been demonstrated very clearly since regular RBCs and their universal chromatic counterparts of analogous composition performed in the same way under a range of static and dynamic mechanical loading regimes and after artificial aging [3]. This analogy and the complexity of the filler systems make it difficult to clarify whether additional fine-tuning of the refractive index within the filler system was carried out in the universal chromatic materials. This could well be assumed since adjusting the refractive index within the filler system and in comparison to the polymer matrix, represents a key mechanism for color and translucency induction in dental resin-based composites [4]. It is also not clearly defined whether pigments are still necessary or only their type or/and quantity have been changed.

The second technology applied to create RBC-UC is more transparent and uses a color generation concept unique to dental materials, inspired by advanced engineering fields and even nature [5]. The so-called structural coloring is achieved through micro- and nanostructured surfaces that are fine enough to interfere with the visible light to enable selective light reflection. The material architecture is built of nano-scaled core-shell structures that entail a material with a higher refractive index (the core) surrounded by a material with a lower refractive index (the shell) [6]. To date, only a single material is available on the market that uses the concept of structural coloring. It consists of a multiscale organization of inorganic-organic core-shell nanoparticles arranged equidistantly, either clustered into round, micrometer-sized fillers with various dimensions and distributions or dispersed in the polymer matrix [1]. In addition to the experimental studies on the microstructure described above, the manufacturer defines the inorganic core as made of silica and zirconia nanoparticles and the organic shell as made of methacrylate polymers based on a mixture of urethane dimethacrylate (UDMA) and triethylene glycol dimethacrylate (TEGDMA). The polymer nature of the shell was confirmed in a recent study that assessed the quasi-static and viscoelastic behavior of the material [1]. The dimension of the silica and zirconia nanoparticles has been shown to have a decisive influence on the color adaptability of the material, with a size of 260 nm proving advantageous [6].

Along with simplifying the restoration procedures and reducing treatment time, developing structural colored dental materials is attractive even from a long-term perspective, as the color instantly adapts to the environment. Treatments, such as teeth bleaching, or age-related tooth color changes should no longer require restoration replacement if the restorative material can adapt to the new situation. While this aspect has not yet been clinically simulated, an enhanced blending effect has indeed been demonstrated [7,8,9], although the latter was dependent on the cavity size [10,11,12]. Further arguments for using structural versus pigment coloring, such as non-fading and less toxic capabilities, have not yet been confirmed in dental materials, as the performance of materials colored by these two methods was similar [1].

The possibility of combining several treatment steps was explored by RBC materials able to be cured in bulk, that means in increments up to 4–5 mm [13]. Such materials can significantly reduce treatment time as they can be applied in single or deeper increments to fill large cavities rather than using multiple thin layers that need to be individually cured. It is accepted nowadays that a bulk-filling technique allows both, reducing the chair time and the risk to induce defects or contaminants between layers, when compared to an incremental placement technique [14]. Whether a bulk-filling technique reduces shrinkage stress development in a RBC restoration is still controversial [15,16,17]. However, it is undeniable that in such materials, an optimal degree of conversion of the polymer matrix is achieved up to a depth of 4–5 mm [18]. Because bulk-fill RBCs come in varying consistencies related to the amount of inorganic reinforcing fillers, the diversity of their mechanical properties [19] dictates the way they are applied clinically. Therefore, restorations with flowable bulk-fill RBCs require an occlusal covering layer of sculptable RBCs, while higher filled bulk-fill materials can be used without capping. Despite concerns about the very low modulus of elasticity of flowable bulk-fill RBCs [19], the described restoration model is clinically successful [20]. Notwithstanding the mentioned limitations, the use of bulk-fill RBCs is spreading.

Bringing new materials to market always requires a thorough investigation of their behavior compared to already clinically proven materials and under simulated clinical conditions, including an aging process. Material comparisons within categories defined mainly by marketing or trends are less relevant in this context. Moreover, the variability in material behavior within such categories turned out to be very high [21]. Therefore, comparing RBC-UCs and RBC classified in shades as material categories is not conducive, as mechanical properties and aging behavior are directly related to the organic/inorganic composition, type, proportion, and micro/nanostructure [21].

The present study aimed to evaluate an innovative, experimental RBC that combines universal chromatic characteristics with the ability to be cured in bulk. In addition, it is a less filled material, but due to a modified rheology, it is still intended for use in posterior occlusal cavities, without necessitating capping with a higher filled RBC. Based on this declared intention for clinical use, a direct comparison is made between clinically successful materials that are higher filled and enable functional modeling of occlusal surfaces and entire cusps. One is a conventional colored RBC which can be applied in bulk up to 4-mm increments [18] and the second is a structural-colored RBC which is applied in 2-mm increments [3]. It is therefore hypothesized that the experimental material performs similarly to clinically proved RBCs in terms of mechanical behavior at (a) macroscopic and (b) microscopic (quasi-static and viscoelastic behavior) levels and (c) after aging.

## 2. Materials and Methods

### 2.1. Materials

An experimental flowable universal chromatic RBC (abbreviated Exp.) was characterized in comparison to a regular bulk-fill micro-hybrid RBC (TPF) and a non-flowable universal chromatic RBC (OC) that have been used clinically for several years (Table 1). 

The aging behavior was assessed on a macroscopic and microscopic level compared to the baseline data recorded 24 h after polymerization. Clinical aging was simulated by alternately immersing the samples 10,000 times between 5 °C and 55 °C distilled water (thermal aging). Characterization at macroscopic level was performed in terms of mechanical strength, modulus, beam deflection, fracture mechanisms, and reliability, while the accelerated aging procedure complemented the standardized test conditions. In addition to the conditions tested at the macroscopic level, more aggressive effects of aging, such as additional storage in ethanol solution after thermal aging, were evaluated at the microscopic and nanoscale levels using a range of parameters to assess changes in both static and viscoelastic behavior.

### 2.2. Three-Point Bending Test

A total of 40 slabs (2 mm × 2 mm × 18 mm) were prepared for each tested RBC according to the recommendation of ISO 4049:2019 [22] for the 3-point bending test. A white polyoxymethylene mold was filled with the uncured material and pressed between two glass plates separated from the material by transparent polyacetate films. The light-curing protocol following ISO 4049:2019 involved irradiation on top and bottom of the specimens with three light exposures overlapping one irradiated section no more than one mm of the diameter of the light guide in order to prevent multiple polymerizations. Exposure time was considered as indicated by the manufacturer, amounting 10 s for TPF and 20 s for the other two materials (Table 1). A blue LED (light-emitting diode) LCU (light-curing unit) was used for polymerization (Bluephase^®^ Style, Ivoclar Vivadent, Schaan, Liechtenstein). The irradiance, radiant exposure, and spectral distribution of the LCU were measured using a spectrophotometer (MARC-RC, Managing Accurate Resin Curing System, Bluelight Analytics Inc., Halifax, NS, Canada).

Immediately after curing, specimens were removed from the mold and were ground with silicon carbide paper (grit size P 1200, Leco Corp. SS-200, Benton Harbor, MI, USA) in order to get rid of disturbing edges or bulges, followed by immersion in distilled water at 37 °C for 24 h in a dark environment. At the end of the immersion time, half of the samples of each material (*n* = 20) underwent a 3-point-bending test, while the other half underwent additional accelerated aging prior to testing (thermal aging, abbreviated TA, 10,000 cycles between 5 °C and 55 °C, dwell time 30 s, transfer time 5 s, Willytec, Dental Research Division, Munich, Germany).

The flexural strength (FS) and flexural modulus (E) were determined in a 3-point bending test according to NIST No. 4877, considering a span of 12 mm [23]. The samples were loaded in a universal testing machine until fracture (Z 2.5 Zwick/Roell, Ulm, Germany) at a crosshead speed of 0.5 mm/min. The force during bending was measured as a function of beam deflection, while the slope of the linear part of this curve was used to calculate the flexural modulus.

### 2.3. Light and Scanning Electron Microscopy (SEM) Evaluation

Fractured surfaces were immediately subjected to fractography analysis under a stereomicroscope (Stemi 508, Carl Zeiss AG, Oberkochen, Germany) to determine fracture pattern and origin according to Quinn’s recommendations [24], and were photographed using a microscope extension camera (Axiocam 305 color, Carl Zeiss AG, Oberkochen, Germany). The origin of fracture was identified as either volume (sub-surface) or surface (edge and corner) defects. For better visualization of the fracture pattern observed in light microscopy, representative patterns were additionally recorded by scanning electron microscopy (SEM, Zeiss Supra 55 V P, Carl Zeiss AG, Oberkochen, Germany). Next, three composite slabs were prepared and cured as described above for each material for microstructure evaluation. The surface of each specimen was wet-finished with a series of silicon carbide abrasive papers (grit size P1200, P2500, and P4000, LECO) and then polished with diamond spray (1 μm; DP-Spray, STRUERS GmbH, Puch, Austria) in an automatic grinding machine (EXAKT 400CS Micro Grinding System, EXAKT Technologies Inc., Oklahoma City, OK, USA). All samples were cleaned ultrasonically (RK 31, Badelin Electronic, Berlin, Germany) for 5 min before scanning electron microscopy evaluation (SEM, Zeiss Supra 55 VP).

### 2.4. Instrumented Indentation Test (IIT): Quasi-Static Approach (ISO 14577 [25])

A further 15 slabs were prepared for each RBC as described above. All samples were initially stored at 37 °C for 24 h in distilled water and then subjected to additional two consecutive aging conditions. One third underwent IIT (*n* = 5) while the rest were submitted to thermal aging (TA) as described previously. Half of the TA samples underwent IIT (*n* = 5) and the other half were additionally aged by immersion in a 75% ethanol/water solution for 3 days previous to measurements (*n* = 5). 

Proximate to measurements, each specimen was wet-ground with silicon carbide paper and polished with a diamond suspension as described above, until the surface was shiny. By means of an automated micro-indenter (FISCHERSCOPE^®^ HM2000, Helmut Fischer, Sindelfingen, Germany) equipped with a Vickers diamond tip, three indentations were performed in each specimen, with a total of 15 indentations for each material and storage conditions. Indentation depth and indentation force were recorded during the whole indentation cycle, with the test force increasing from 0.4 mN to 1000 mN, followed by a holding time of 5 s at maximum force and a subsequent reduction in force within 20 s at a constant speed. Change in indentation depth recorded during holding time served as a measure for the material’s creep (C_IT_). The integral of the indentation force over the depth defines the mechanical work W_total_ (= ∫Fdh), which is partially consumed as plastic deformation work W_plast_, while the rest is released as work of the elastic recovery W_elastic_. Indentation creates an impression with projected indenter contact area (A_c_) determined from the force-indentation depth curve considering the indenter correction based on the Oliver and Pharr model and described in ISO 14577 [25]. Therefore, the indenter area function was calibrated to two different materials with uniform and known material properties (sapphire and quartz glass). Corrections obtained from the tip calibration were then used for further computational data analysis. The indentation hardness (H_IT_ = F_max_/A_c_) is a measure of the resistance to plastic deformation and is convertible to the more familiar Vickers hardness (HV = 0.0945 × H_IT_). The universal hardness (or Martens hardness = F/A_s_(h)) was calculated by dividing the test load by the surface area of the indentation under the applied test load (As), giving a characterization of both plastic and elastic deformation. The indentation modulus (E_IT_) was calculated from the slope of the tangent of the indentation depth-curve at maximum force.

### 2.5. Instrumented Indentation Test (IIT); Dynamic Mechanical Analysis (DMA)

The DMA test used a low-magnitude oscillating force (10 different frequencies in the range 0.5–5 Hz) that was superimposed onto a quasi-static force of 1000 mN. The oscillation amplitude was set at 5 nm so that the sample deformation was kept within the linear viscoelastic regime. Six randomly chosen indentations were performed per specimen, amounting 30 individual indentations per RBC brand and aging condition, while ten repeated measurements were performed for each frequency and indentation. 

For the used frequency, the force oscillation generates oscillations on the displacement signal with a phase angle δ. The sinusoidal response signal was then separated into a real part and an imaginary part representing the storage (E′) and the loss moduli (E″), respectively. E′ is a measure of the elastic response of a material behavior, whereas E″, characterizes the viscous material behavior. The quotient E″/E′ is defined as the loss factor (tan δ) and is a measure of the material damping behavior.

### 2.6. Statistical Analysis

All variables were normally distributed, allowing a parametric approach to be used. Multifactor analysis of variance was applied to compare the parameters of interest (flexural strength; flexural modulus; Martens, Vickers, and indentation hardness; elastic and total indentation work; creep; indentation depth; storage, loss, and indentation moduli; loss factor). The results were compared using one- and multiple-way analysis of variance (ANOVA) and Tukey honestly significant difference (HSD) post-hoc test using an alpha risk set at 5%. A multivariate analysis (general linear model) assessed the effect of parameters *material*, *aging*, and *frequency* as well as their interaction terms on the analyzed properties. The partial eta-squared statistic reported the practical significance of each term based on the ratio of the variation attributed to the effect. Larger values of partial eta-squared (η_P_^2^) indicate a higher amount of variation accounted for by the model (SPSS Inc. Version 27.0, Chicago, IL, USA).

Flexural strength data were additionally described by a Weibull analysis. A common empirical expression for the cumulative probability of failure P at applied stress σ is the Weibull model [26]:

$$P_{f}(\sigma_{c})=1-\exp\left[-{\left(\frac{\sigma_{c}}{\sigma_{0}}\right)}^{m}\right]$$

where $\sigma_{c}$ is the measured strength, m the Weibull modulus and $\sigma_{0}$ the characteristic strength, defined as the uniform stress at which the probability of failure is 0.63. The double logarithm of this expression gives:$\ln\ln\frac{1}{1-P}=m\ln\sigma_{c}-m\ln\sigma_{0}$. By plotting ln ln(1/(1 − *P*)) versus ln $\sigma_{c}$, a straight line results in the upward gradient m, whereas the intersection with the x-axis gives the logarithm of the characteristic strength [26].

## 3. Results

### 3.1. Three-Point Bending Test

**Figure 1:**
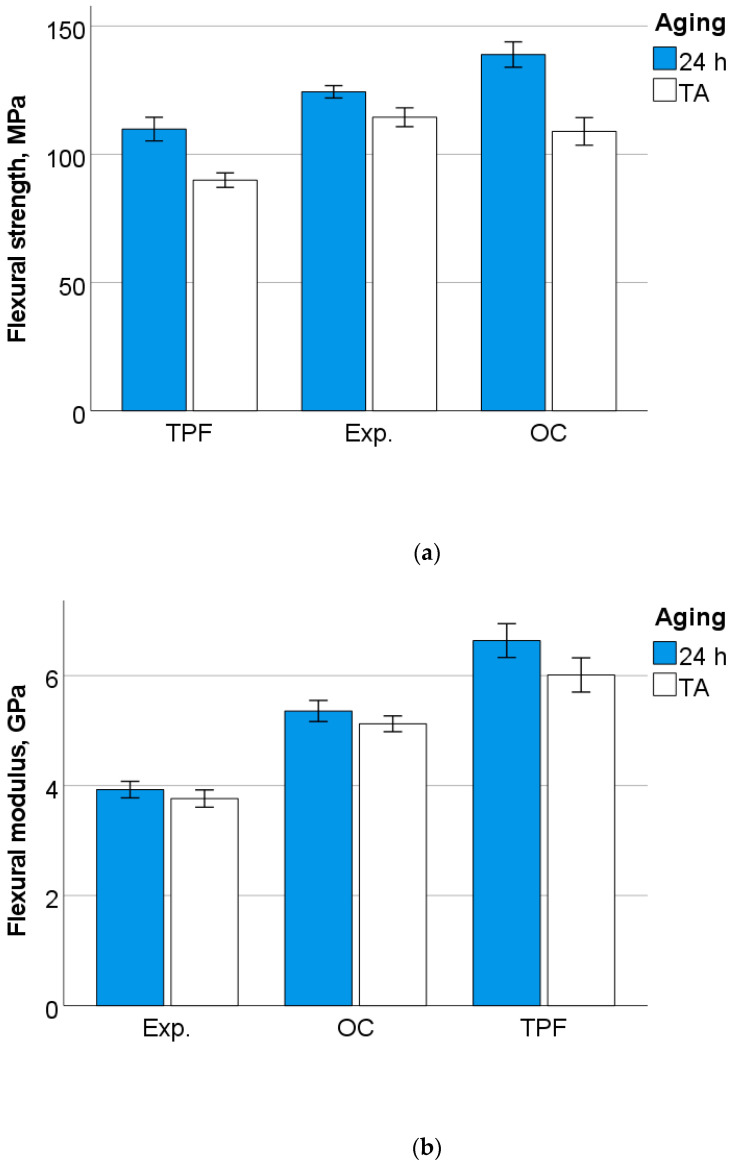

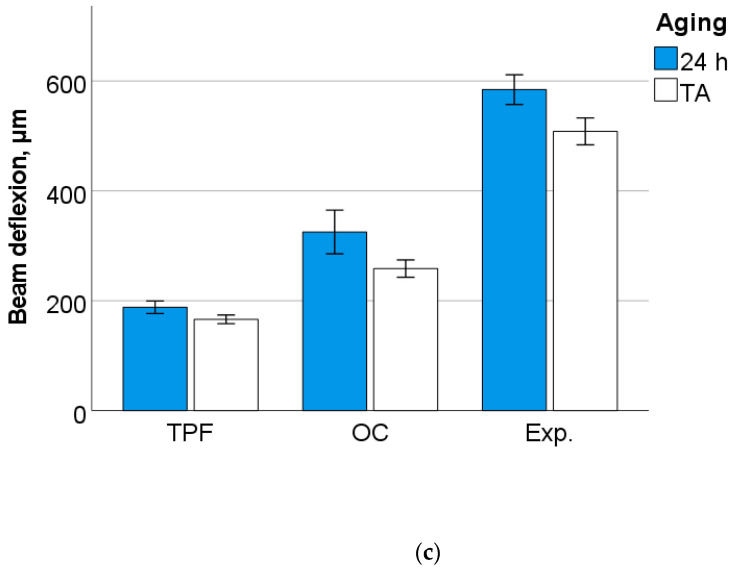
Outcome of the 3-point bending test: (**a**) flexural strength, (**b**) flexural modulus, and (**c**) beam deflection as a function of material and aging, presented in ascending order of the data for unaged (24 h post-polymerization) specimens. TA, thermal aging. Mean values and the 95% confidence are indicated.

**Figure 2:**
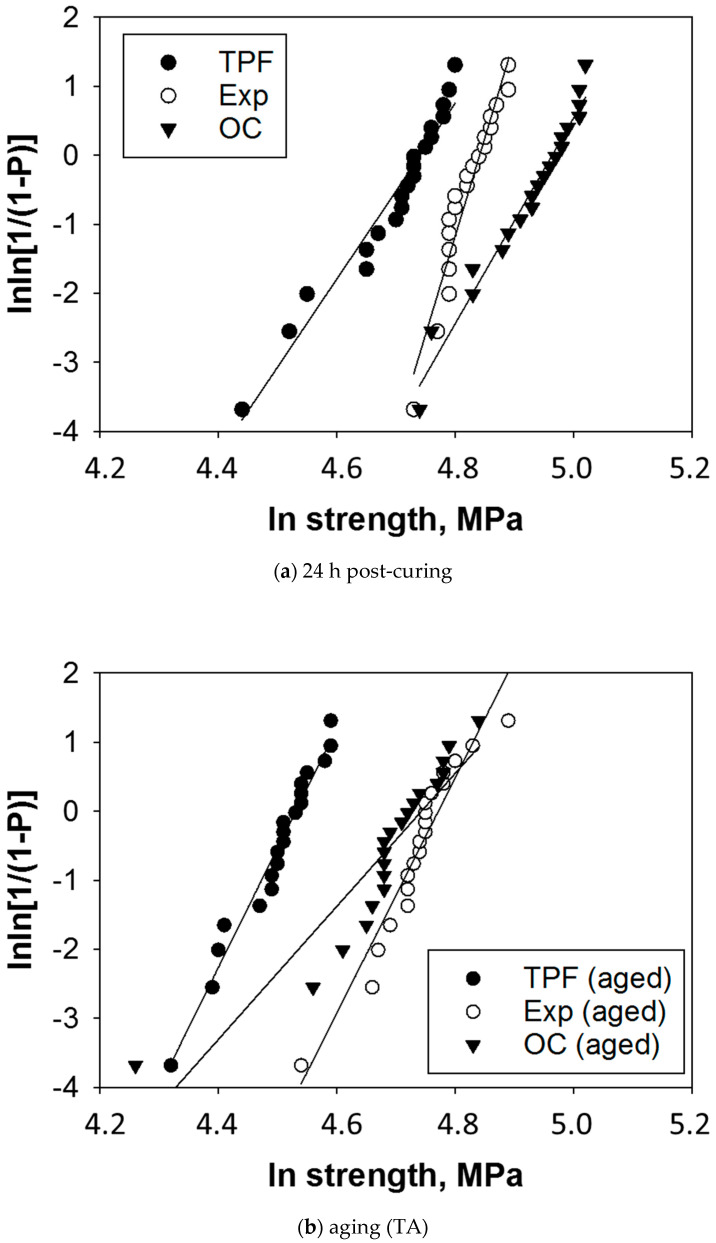
Weibull plot representing the empirical cumulative distribution function of strength data per material and aging conditions. Linear regression was used to numerically assess the goodness-of-fit and estimate the parameters of the Weibull distribution in (**a**) 24 h post-irradiated and (**b**) thermal aged specimens (Table 2).

**Figure 3:**
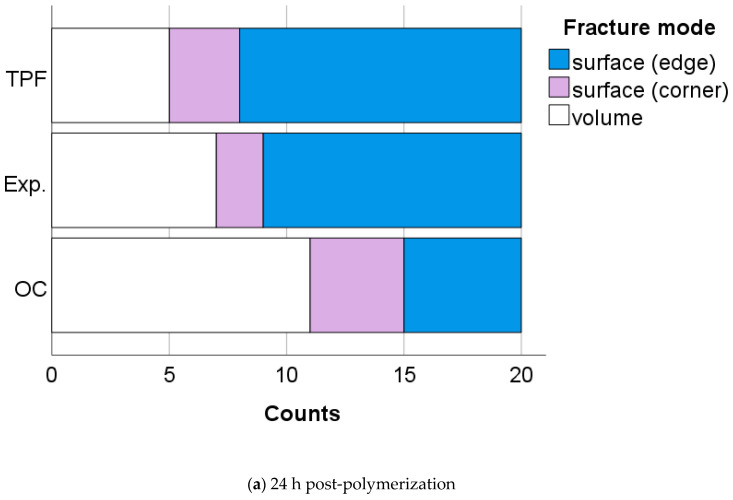

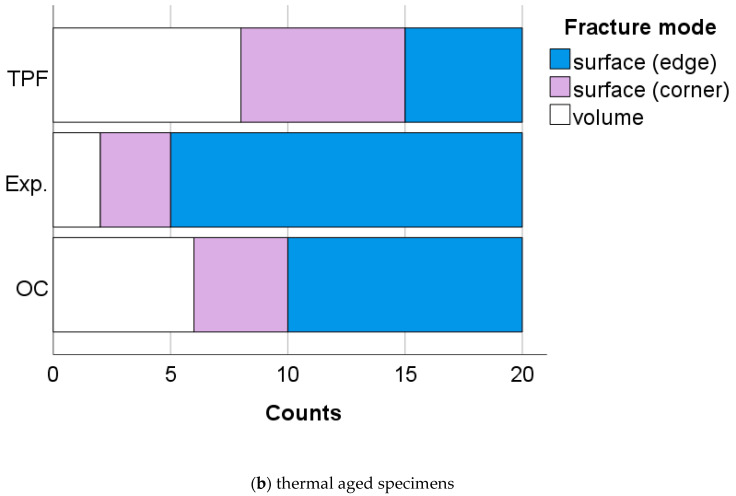
Fracture mode as a function of material and aging (**a**) 24 h post-polymerization and (**b**) thermal aged.

**Figure 4:**
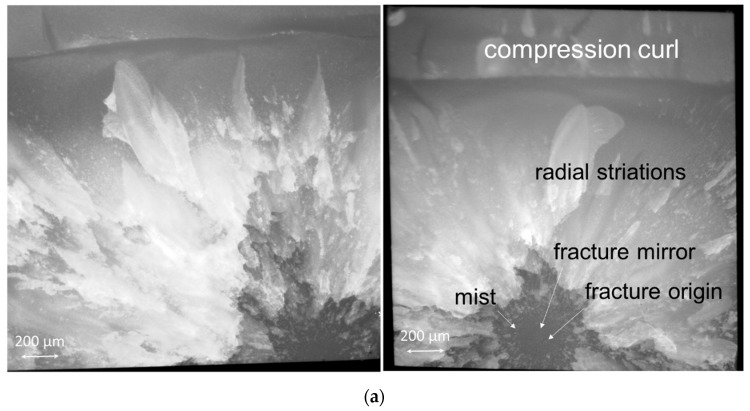

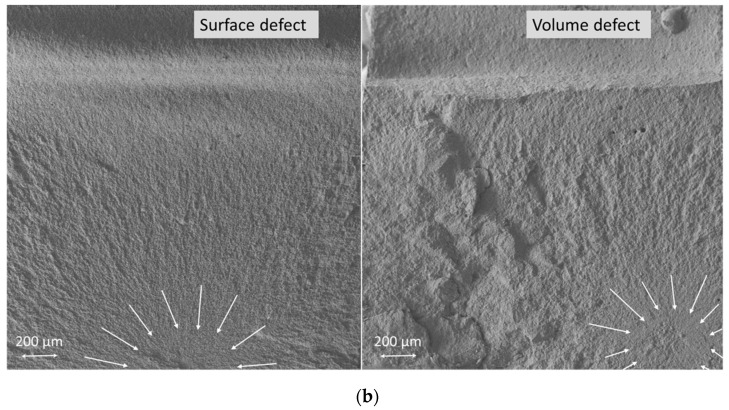
(**a**) Stereo microscopy and (**b**) scanning electron microscopy images illustrating the fracture pattern and the fracture origin. Left: surface defect (localized on the edge of the fractured images); Right: volume defect (sub-surface). The fracture mirror, which is the smooth surface in the initial part of the fracture created when the fracture is accelerated is demarked by the arrows pointing towards the fracture origin. The rougher surface adjacent to the mirror (mist) is followed by crack propagation in different directions, resulting in radial striations (Hackle lines), while the upper part of the image depicts the compression curl confirming the final breakthrough. The image shows the entire 2 mm × 2 mm fractured area, while the tensile zone is located in the lower part of each image.

The outcome of the 3-point bending test is summarized in Figure 1, Figure 2, Figure 3 and Figure 4. A multifactorial analysis showed a significant effect of the factors *material* and *aging* (*p* < 0.001) on all measured parameters except for the fracture mode. Their binary combined effect was significant only for the flexural strength (*p* < 0.001, η_P_^2^ = 0.185). For all measured parameters, the influence of the *material* was stronger than the influence of *aging*. The *material* most affected the beam deflection (*p* < 0.001; η_P_^2^ = 0.908), followed by flexural modulus (η_P_^2^ = 0.828) and flexural strength (η_P_^2^ = 0.596). The descending order in the effect of *aging* was calculated as flexural strength (η_P_^2^ = 0.574) > deflection (η_P_^2^ = 0.238) > flexural modulus (η_P_^2^ = 0.119).

In unaged specimens, one-way ANOVA clearly distinguishes the data into three homogeneous subgroups (*p* < 0.001) in ascending order, as shown in Figure 1a–c. The same applies to aged specimens, with the amendment that the flexural strength of OC and Exp. were statistically similar (*p* = 0.124).

The reliability of the flexural strength data for analyzed materials and aging conditions was evaluated by a Weibull statistic (Figure 2, Table 2). High R^2^ values are observed for all groups (R^2^ > 0.92), indicating a very good fit of the Weibull model, except for the thermal aged OC group, where the coefficient of determination of the regression analysis was slightly lower (0.86). The lower fit of the regression analysis to this data was caused by a single specimen with a lower strength value (70.9 MPa), which was not excluded from the statistical analysis, as can be observed in Figure 2. The 95% confidence interval of the Weibull modulus m can be assessed from the indicated standard error (subtracting and adding 1.96 times the standard error from the mean indicated in Table 2). Within the 95% confidence interval, a significantly higher m-value was calculated in the experimental material compared to the other two groups, which performed statistically similarly. After aging, all materials, except TPF, showed a reduced Weibull modulus. This variation resulted in equal m-values for TPF and the experimental material, both higher than OC.

The fracture mode is presented in Figure 3a,b. In unaged specimens (Figure 3a), the highest percentage of failures came from surface defects (46.7% edge and 15.0% corner), while 38.3% of the failure was initiated from volume defects. This relation persists after accelerated aging, with the exact percentage being 50.0%, 23.3%, and 26.7%, respectively (Figure 3b).

The specific failure modes presented above impart characteristic features to the fractured surface, as exemplified in Figure 4, showing fractures originating from surface defects located at the edge (a) and corner as well as volume defects (b).

**Figure 5:**
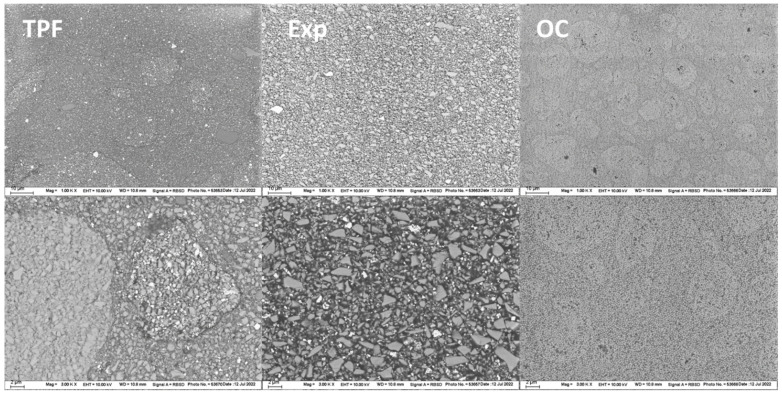
SEM-images: Structural appearance of the filler system, measured in the electron backscatter diffraction mode; TPF = Tetric PowerFill, Exp = experimental bulk-fill universal chromatic RBC, OC = Omnichroma, in two magnifications: 1000× (upper picture; scale = 10 μm) and 3000× (lower picture; scale = 2 μm).

The structural appearance of the filler system is illustrated in Figure 5. Because images were taken in electron backscatter diffraction mode, fillers of different compositions appear whitish (higher atomic number, e.g., ZrO_2_, YbF_3_) or darker (lower atomic number, e.g., SiO_2_, polymer), allowing differentiation between individual parts of the complex filler system.

Two different types of pre-polymerized fillers can be clearly distinguished in TPF at the higher magnification. One type looks similar in composition to the material in which it is embedded, is clearly demarcated as a filler, and appears to contain a higher amount of fillers with high atomic number (white), which are also dispersed throughout the rest of the composite. The other pre-polymerized filler type appears to be more homogeneous in the composition containing only one type of inorganic filler dispersed in an organic polymer matrix. The lower magnification additionally reveals compact glass fillers (grey) with sizes below 5 µm together with the whitish smaller fillers mentioned above. In contrast, no pre-polymerized fillers were identified in Exp., but compact glass fillers with sizes below 2 µm, along with tiny fillers containing elements of high atomic number, as they appear whitish. A completely different microstructure is observed in OC, where polymer-coated SiO_2_ and ZrO_2_ nano-fillers are clustered into larger round fillers of several µm. The matrix in which these larger fillers appear to be embedded consists of a polymer in which SiO_2_ and ZrO_2_ nano-fillers are dispersed, as seen in the higher magnification. Interestingly, the spacing between the nano-fillers appears to be similar, either when grouped in the round, larger, distinct fillers, or in what appears to be the matrix.

### 3.2. Instrumented Indentation Test (IIT): Quasi-Static Approach

A multifactorial analysis indicates a significant (*p* < 0.001) and very strong effect of both parameters *material* and *aging*, while the effect of *material* was, similar to what was observed for the macro-mechanical properties, stronger as the *aging*. *Material* influenced strongest HM (η_P_^2^ = 0.971), HV (η_P_^2^ = 0.970), E_IT_ (η_P_^2^ = 0.969), followed by W_t_ (η_P_^2^ = 0.963), C_IT_ (η_P_^2^ = 0.962), W_e_ (η_P_^2^ = 0.938), and n_IT_ (η_P_^2^ = 0.905). As for *aging*, it affected strongest W_e_ (η_P_^2^ = 0.868), followed by HM (η_P_^2^ = 0.863), W_t_ (η_P_^2^ = 0.861), E_IT_ (η_P_^2^ = 0.851), HV (η_P_^2^ = 0.848), n_IT_ (η_P_^2^ = 0539), and C_IT_ (η_P_^2^ = 0.244). The binary combination of the effects of *material* and *aging* was also significant on all measured parameters (*p* < 0.001).

**Figure 6:**
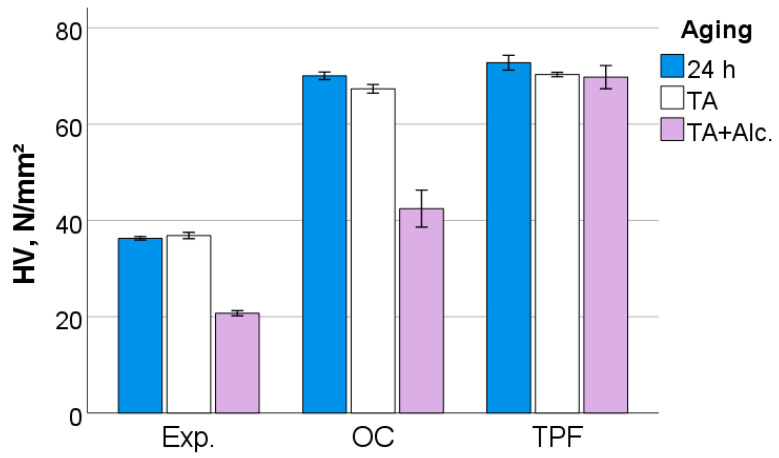
Vickers hardness (HV) as a function of RBC and aging conditions (mean values with 95% confidence interval); 24 h = 24 h post-irradiation, TA = thermal aging, TA+Alc. = thermal aging followed by storage for 3 days in alcohol solution.

**Figure 7:**
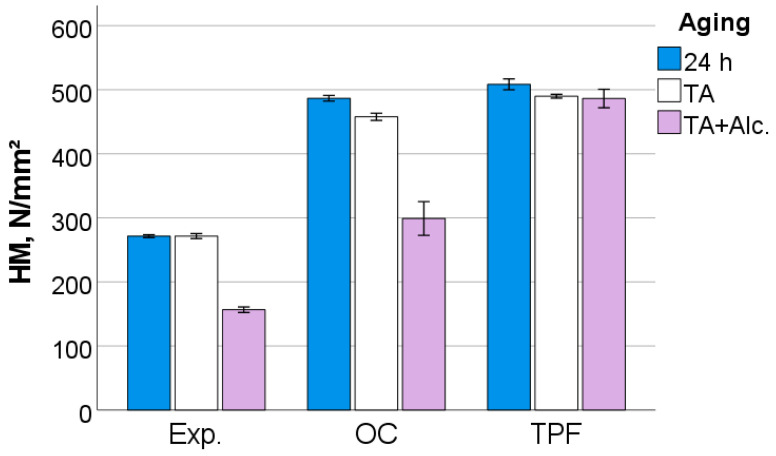
Martens hardness (HM) as a function of RBC and aging conditions (mean values with 95% confidence interval).

**Figure 8:**
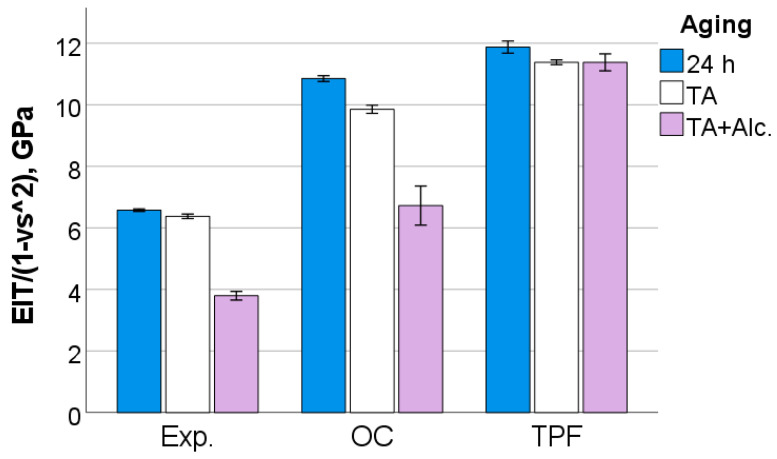
Indentation modulus as a function of RBC and aging conditions (mean values with 95% confidence interval).

**Figure 9:**
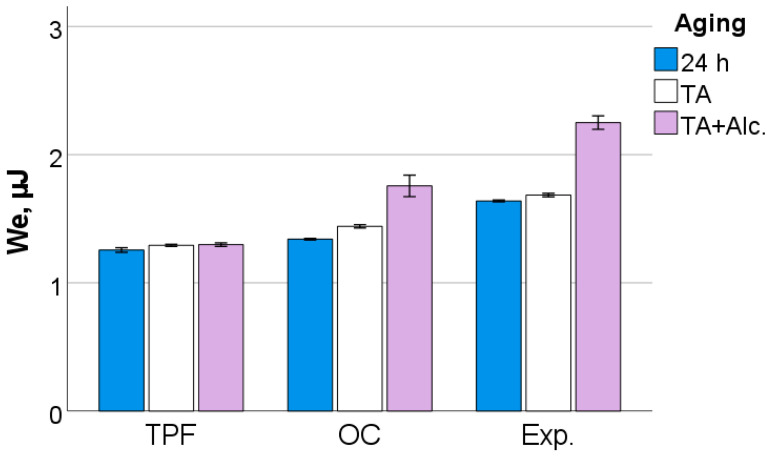
Elastic indentation work as a function of RBC and aging conditions (mean values with 95% confidence interval).

**Figure 10:**
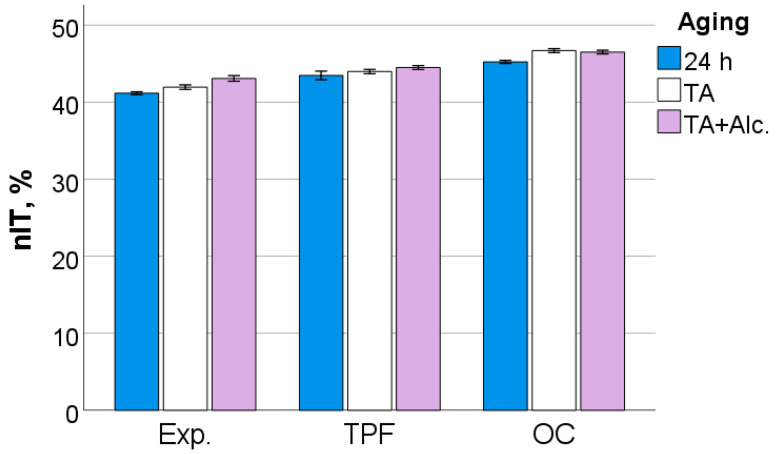
Elastic indentation work related to the total indentation work as a function of RBC and aging conditions (mean values with 95% confidence interval).

**Figure 11:**
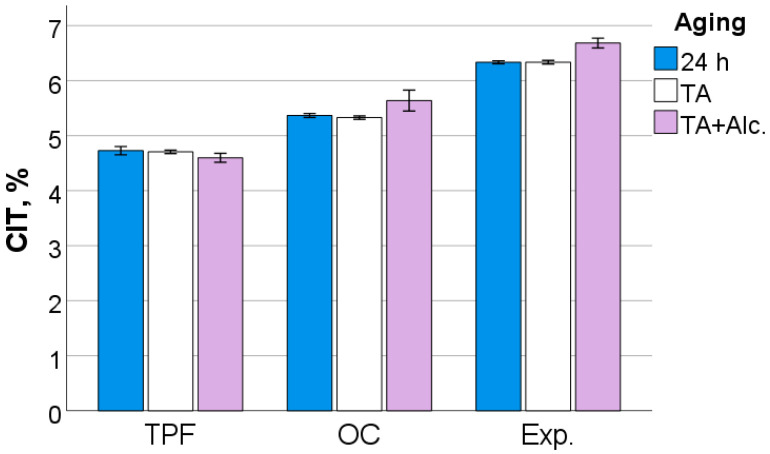
Creep as a function of RBC and aging conditions (mean values with 95% confidence interval).

After 24 h following polymerization, there is a significant difference (*p* < 0.001) between analyzed materials in all measured micromechanical properties. The ascending material sequence for HV, HM and E_IT_, as also envisaged in the figures below, was Exp. < OC < TPF. For C_IT_ and W_e_, the order is similar but descending: Exp. > OC > TPF. The only exception to the ranking was observed for n_IT_ and was Exp. < TPF < OC. The statistically significant difference (*p* < 0.001) between materials and the sequence is retained after thermal aging (TA) and additional storage in alcohol solution. Figure 6, Figure 7, Figure 8, Figure 9, Figure 10 and Figure 11 summarize the data described above, with the mean and 95% confidence interval being indicated.

### 3.3. Instrumented Indentation Test (IIT); Dynamic Mechanical Analysis (DMA)

A multifactorial analysis shows that the analyzed factors, i.e., *material*, *aging,* and *frequency*, had a significant (*p* < 0.01) influence on all the measured parameters (*p* < 0.001). Their binary and ternary combinations also significantly affected all parameters except for H_IT_ when the binary and ternary combinations involved the parameter *frequency*. The effect of *material* was very strong on H_IT_ (η_P_^2^ = 0.968) and storage modulus (η_P_^2^ = 0.949), while moderate on the viscoelastic parameters loss factor (η_P_^2^ = 0.545) and loss modulus (η_P_^2^ = 0.312). In comparison, the effect strength of *aging* varies in the same sequence, but was lower (H_IT_, η_P_^2^ = 0.811; storage modulus, η_P_^2^ = 0.664; loss factor, η_P_^2^ = 0.337; and loss modulus, η_P_^2^ = 0.229). The *frequency* exerted a low influence on H_IT_ (η_P_^2^ = 0.155) and a moderate to high effect on the other parameters (storage modulus, η_P_^2^ = 0.860; loss modulus, η_P_^2^ = 0.764; loss factor, η_P_^2^ = 0.633).

**Figure 12:**
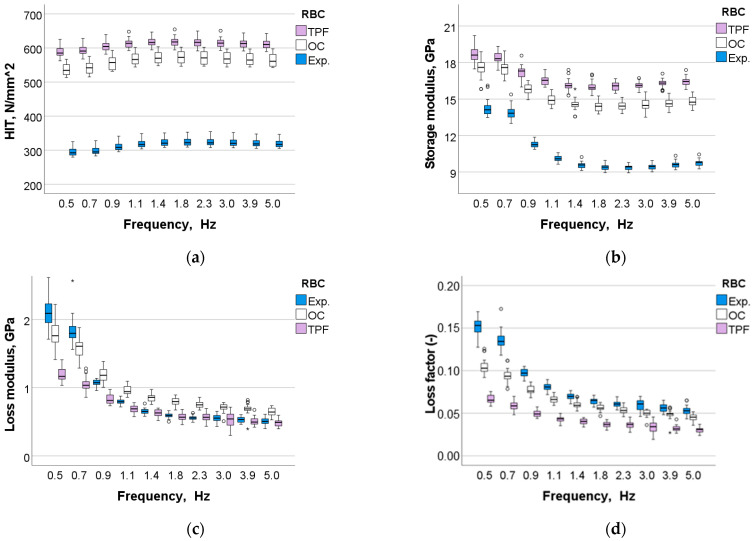
Dynamic mechanical analysis: (**a**) indentation hardness, (**b**) storage modulus, (**c**) loss modulus and (**d**) loss factor as a function of material and frequency.

**Figure 13:**
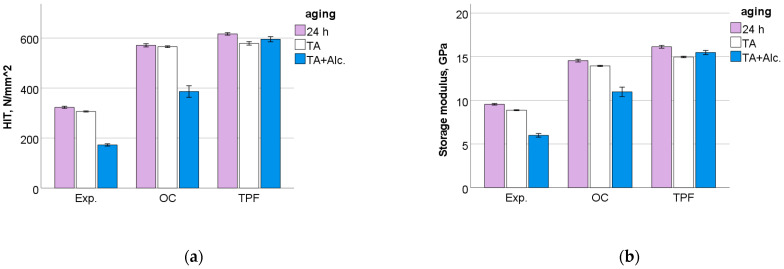

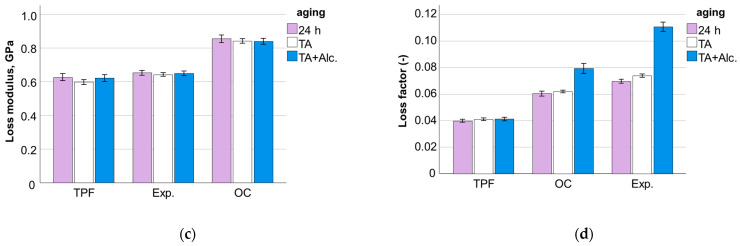
Variation of the (**a**) indentation hardness, (**b**) storage modulus, (**c**) loss modulus and (**d**) loss factor as a function of material and aging conditions exemplified for a frequency of 1.4 Hz (mean values with 95% confidence interval).

The pattern of variation of the analyzed parameters with frequency was similar for all materials and aging conditions but differed between parameters. H_IT_ shows a slight increase up to a frequency of 1.1 Hz and hold these values for the higher frequency range tested. In contrast, the other parameters showed a steady decrease with frequency, up to 1.4 Hz. The measured values for H_IT_ and storage modulus rank the materials in the same order as previously observed for the quasi-static parameters, namely Exp. < OC < TPF, while the ranking for the viscoelastic parameters was reversed (Figure 12a–d). Figure 13a–d depict the influence of aging on the measured parameters and materials for a frequency of 1.4 Hz, which is in the range of chewing frequency in humans.

## 4. Discussion

The experimental material analyzed in this study joins two main modern research trends in the development of dental RBCs towards simplified clinical application. It combines the now well-accepted placement of RBCs in bulk with efforts at universal chromatic or one shade coloring. It is formulated as a flowable RBC with a modified rheology that should ensure sufficient stability to be used as a sculptable RBC without capping when placed in posterior cavities.

As the experimental material is intended to be used in posterior occlusal cavities, the flexural strength measured in a standardized three-point bending test according to ISO 4049 is considered as one of the most important parameters to be monitored [22]. ISO 4049 stipulates for materials intended to be used in occlusal areas a flexural strength larger 80 MPa [22], a value which was exceeded by far in all analyzed materials, including Exp. While ISO 4049 [22] only specifies the minimum flexural strength measured 24 h post-polymerization, aging can significantly reduce strength. In fact, thermal aging has registered a slight deterioration in the flexural strength for all materials, but the values still remained above the limit specified in the standard [22]. Because the measurements were performed in a standardized manner [22], data can be compared not only within the study design, but also with data compiled over time in a large database that includes many clinically successful materials [21]. With the deliberate criticism of material comparisons in categories presented in the introduction, currently used nano-hybrid RBCs for posterior restorations show an average flexural strength of (103.1 ± 19) MPa and a large scatter of the values among the materials [21]. This association indeed allows the conclusion that Exp. can be compared well with clinically established materials [21]. However, the flexural strength data needs to be backed up with another important parameter, the flexural modulus, which unfortunately is not accounted for in ISO 4049 [22], exposing the standard to intense criticism in the dental materials community. This criticism is supported by the clear observation that many flowable materials with low filler content, which show high deflection to failure, can lead to high flexural strength values [21], although they are not indicated in large cavities in the posterior region. Fortunately, the flexural modulus is recorded and reported in many studies [21], which in turn allows comparison with large databases [21]. For the data set used to compare flexural strength, the mean flexural modulus was (5.0 ± 0.8) GPa [21], placing Exp. at the lower limit of the interval, with a modulus of 4 GPa. The other two materials tested have a significantly higher modulus (5.3 to 6.6 GPa), which is included in the specified interval [21]. This behavior is consistent with the amount of inorganic filler being lowest in Exp., as indicated in Table 1.

On the positive side, however, the reliability expressed by the Weibull modulus m was highest in Exp., which may be an effect of a more homogeneous distribution of the filler and the microstructure (Figure 5 Exp.). Since all specimens were ground after demolding, this effect cannot be attributed to the lower viscosity and thus better adaptation to the mold when inserting the unpolymerized paste. SEM analysis confirms this assumption as TPF contains a wider variety of fillers, including pre-polymerized and compact fillers. Apart from the different size and morphology of the fillers, pre-polymerized fillers can be bound to the polymer matrix to a different extent than the compact glass fillers (Figure 5 TPF). While porosities are inevitable in resin composites, in OC they can be evident both within the round fillers that encompass the nanostructured core-shell silica and zirconia fillers, and the matrix, defined as the space between these fillers (Figure 5 OC). This may be a reason for the higher amount of fracture initiated from volume defects in OC samples 24 h after polymerization, while fracture in the other two materials was more frequently caused by surface defects. On the other hand, the amount of fracture induced by surface defects is often related to the filler size [27], since larger fillers that are pulled out when the specimen is ground or are not bonded strongly enough in the organic matrix may represent defects that can initiate fracture. Fracture mechanisms changed slightly during aging in the analyzed materials, with plasticization of the organic matrix contributing to the microstructural effect of the material. The change in fracture mechanism observed after thermal aging may well account for the greater decrease of FS data within a material after aging, while this effect on the flexural modulus, measured in the initial and linear parts of the force–displacement diagram, is less sensitive to surface changes induced by aging.

The difference in the amount of inorganic filler is also clearly seen in the quasi-static parameters, with the materials being ranked similarly to the macroscopic parameters FS and E. As the measurements occurred at shallower depths (indentation depths varied from 9 µm to 13 µm), they are less affected by large defects such as voids or porosities. Thermal aging showed a significant but small effect on all materials, confirming data measured at the macroscopic level for E. Hydrolytic degradation is a characteristic of methacrylate-based RBC restorations [28] and is observed in various methacrylate-based matrix formulations [29], since the monomers used in dental materials contain a variety of chemical bonds such as esters, ethers, urethanes and amides that can be cleaved by hydrolysis. This effect is amplified by the degradation of the silanes that couple the fillers with the organic matrix, since the silanes bound to the fillers can be hydrolyzed, causing detachment from the matrix, while in addition also the alkoxy part of the amphiphilic silane molecules can be broken down by the mechanisms mentioned above [30]. 

In contrast to TA, the additional storage in alcohol solution, which represents a quite aggressive aging condition, has a great and differentiated impact. Interestingly, TPF showed only a slight decline of properties after alcohol aging, which may be accounted to the higher amount of urethane monomers in the organic matrix. Although it is the only material tested with no declared TEGDMA as a diluent monomer in the composition of the organic matrix, this simple explanation would be quite speculative. To compensate for TEGDMA, a number of low viscosity monomers were apparently used, such as TCDDD and bis-EMA. More plausible in this context would be the significantly lower proportion of the organic matrix prone to be plasticized when immersed in solvent, since the proportion of inorganic fillers was highest in this material (Table 1). Although the amount of filler reported for OC appears to be the highest in the range of materials analyzed (Table 1), this value does not refer to the inorganic, but to the total amount of filler. Considering that the silica and zirconia particles are surrounded by a polymer shell, the de facto proportion of inorganic filler is significantly lower. This was undeniably reflected in the lower flexural modulus and IIT parameters in OC compared to TPF.

We chose to perform the DMA analysis at frequencies relevant to human chewing activity, estimated between 0.94 Hz and 2.17 Hz, similar to current study designs for dental materials [3]. The variation of the measured parameters with frequency is consistent with the data measured in the quasi-static and 3-point bending tests and confirms the assumption of a higher inorganic filler amount in TPF vs. OC. While H_IT_ shows a fairly similar pattern of variation across all materials, the storage modulus decreases with frequency the faster the higher the organic fraction in the material (Table 1). Interestingly, frequencies above 0.9 Hz were more discriminatory in this respect than lower ones, as the OC and TPF comparison clearly shows. In general, the faster a stress is applied, the less time the molecules have to relax and absorb that stress [31]. It thus indicates a better adaptation of TPF to variable frequencies than OC or even more Exp., which has the highest amount of polymer in its composition (Table 1).

In contrast to the storage modulus, the parameters characterizing the viscous behavior, loss modulus and loss factor, decreased exponentially with frequency at a more homogeneous rate among materials. The viscos behavior in RBCs must be related to internal friction generated by phase boundary relaxation [32] and molecular rearrangements by polymer chain relaxation in response to an applied stress. The loss modulus was highest in OC, but only for frequencies greater than 0.9 Hz, which must be related to the particular microstructure of the material, since the proportion of organic matrix was lower compared to Exp. (Table 1). Since OC consists exclusively of nano-fillers (Figure 5), the structure offers a very large filler/matrix interface at which stress can be relieved [32], which explains the atypical behavior. This microstructure was chosen primarily for structural color intent, since scattering in a RBC is greatest when the filler diameter approaches approximately half the wavelength of the incident light, i.e., ~0.2–0.3 µm [4], a range that fits well with the particle size of the silica and zirconia nano-fillers in OC (0.260 µm). On the other hand, the loss factor characterizes the damping behavior of a material [32] and thus its ability to withstand mechanical stresses that occur, e.g., during chewing. It was highest in the experimental material due to the higher polymer content and unfortunately confirms many other investigations, since storage modulus and loss factor are mutually exclusive [3]. An ideal dental material should have both high storage moduli and high damping capacity, a desire that is difficult to achieve in the cured systems.

Consequently, the defined null hypotheses must all be rejected since the performance of the experimental material differs from the reference materials. In addition, the reference materials perform differently in a direct comparison on a macroscopic–microscopic level and after artificial aging.

## 5. Conclusions

The materials examined show clear differences in their behavior in all tested parameters as well as after accelerated aging, which can be attributed to the filler content, chemical composition, and microstructure. The experimental universal chromatic bulk-fill RBC, even after aging, exceeds the ISO 4049 flexural strength threshold for materials to be placed in posterior cavities. Its lower inorganic filler amount is directly reflected in lower elastic moduli on the macro and micro scale compared to clinically successful reference materials, somewhat limiting placement in posterior areas to smaller cavities.

## Figures and Tables

**Table 1:** Analyzed RBCs and chemical composition as indicated by the manufacturer.

Code	RBC/Manufacturer	Shade	LOT	Curing Time	Monomer	Filler	
						Composition	wt/vol%
**Exp.**	experimental (Venus Bulk Flow One) Kulzer	one	M010021	20 s	Bis-GMA, UDMA, TEGDMA E(4)BADMA	Ba-Al-B-F-Si glass, SiO_2_, YbF_3_	65/41
**TPF**	Tetric PowerFill Ivoclar Vivadent	^IV^A	Z02XGF	10 s	Bis-GMA, UDMA, TCDDD, bis-EMA	Ba-Al-B-F-Si glass, PPF, SiO_2_, YbF_3_	79/53–54
**OC**	Omnichroma Tokuyama	one	064B2	20 s	UDMA, TEGDMA	SiO_2_, ZrO_2_	79/68

Abbreviations: Bis-GMA = bisphenol A glycol dimethacrylate; TEGDMA = triethylene glycol dimethacrylate; E(4)BADMA = ethoxylated bisphenol A dimethacrylate; UDMA = urethane dimethacrylate; TCDDD = tricyclodecane dimethanol dimethacrylate; SiO_2_ = silicon oxide (silica); ZrO_2_ = zirconium oxide (zirconia); YbF_3_ = ytterbium fluoride; PPF = prepolymerised fillers.

**Table 2:** Weibull parameter (m) with standard error (SE) and coefficient of determination of the regression model (R^2^) within each material and aging.

Aging	RBC	R^2^	m	SE
24 h	TPF	0.95	12.7	0.70
	Exp	0.93	28.5	1.89
	OC	0.97	14.9	0.64
aged	TPF	0.97	17.4	0.74
	Exp	0.92	17.0	1.19
	OC	0.86	9.6	0.93

## Data Availability

The datasets generated during and/or analysed during the current study are available from the corresponding author upon reasonable request.
